# ACOX-driven peroxisomal heterogeneity and functional compartmentalization in brown adipocytes of hypothyroid rats

**DOI:** 10.1098/rsos.230109

**Published:** 2023-05-03

**Authors:** Marija Aleksic, Igor Golic, Aleksandra Jankovic, Aleksandra Cvoro, Aleksandra Korac

**Affiliations:** ^1^ Center for Electron Microscopy, Faculty of Biology, University of Belgrade, Belgrade 11000, Serbia; ^2^ Institute for Biological Research 'Sinisa Stankovic'—National Institute of Republic of Serbia, University of Belgrade, Belgrade 11000, Serbia

**Keywords:** hypothyroidism, brown adipocytes, peroxisomes, ACOX1, ACOX3

## Abstract

We previously demonstrated that hypothyroidism increases peroxisomal biogenesis in rat brown adipose tissue (BAT). We also showed heterogeneity in peroxisomal origin and their unique structural association with mitochondria and/or lipid bodies to carry out β-oxidation, contributing thus to BAT thermogenesis. Distinctive heterogeneity creates structural compartmentalization within peroxisomal population, raising the question of whether it is followed by their functional compartmentalization regarding localization/colocalization of two main acyl-CoA oxidase (ACOX) isoforms, ACOX1 and ACOX3. ACOX is the first and rate-limiting enzyme of peroxisomal β-oxidation, and, to date, their protein expression patterns in BAT have not been fully defined. Therefore, we used methimazole-induced hypothyroidism to study ACOX1 and ACOX3 protein expression and their tissue immunolocalization. Additionally, we analysed their specific peroxisomal localization and colocalization in parallel with peroxisomal structural compartmentalization in brown adipocytes. Hypothyroidism caused a linear increase in ACOX1 expression, while a temporary decrease in ACOX3 levels is only recovered to the control level at day 21. Peroxisomal ACOX1 and ACOX3 localization and colocalization patterns entirely mirrored heterogeneous peroxisomal biogenesis pathways and structural compartmentalization, e.g. associations with mitochondria and/or lipid bodies. Hence, different ACOX isoforms localization/colocalization creates distinct functional heterogeneity of peroxisomes and drives their functional compartmentalization in rat brown adipocytes.

## 1. Introduction

Peroxisomal fatty acid oxidation plays an important role in lipid metabolism ensuring lipid turnover homeostasis. The first and rate-limiting enzyme in peroxisomal β-oxidation is acyl-CoA oxidase (ACOX, EC. 1.3.3.6) [1]. There are three mammalian ACOX isoforms, localized in the peroxisomal matrix and highly specific toward their substrates: ACOX1 (palmitoyl-CoA oxidase), ACOX2 (cholestanoyl-CoA oxidase) and ACOX3 (pristanoyl-CoA oxidase) [1–4].

In rats, ACOX2 is present in liver peroxisomes, interacting with bile acid intermediates, whereas ACOX1 and ACOX3 are found in extrahepatic peroxisomes. ACOX1 is active against CoA esters: straight-chain monocarboxylic and dicarboxylic fatty acids, prostaglandins, very-long-chain fatty acids and xenobiotics. ACOX3 is active against 2-methyl-branched fatty acids, long-chain fatty acids and very-long-chain fatty acids [5]. Only ACOX1 is induced by peroxisomal proliferators and its transcription is controlled by PPAR*α* through a specific response element [2,5].

In humans, only two acyl-CoA oxidases were originally identified: ACOX1 with a substrate specificity similar to that seen in rats (a palmitoyl-CoA oxidase) and ACOX2 which oxidizes 2-methyl-branched fatty acids, very-long-chain fatty acids and bile acid precursor esters [5]. A rat ACOX3 homologue was later found in humans but its protein expression has not been detected in tissues and it is considered to be dysfunctional [6].

Peroxisomal β-oxidation is particularly important in brown adipose tissue (BAT), the main thermogenic organ, where fatty acids are substrates for the oxidation process and heat production by non-shivering thermogenesis [7]. Both cold exposure and a high-fat diet increase peroxisomal abundance via thermogenic activation of BAT [8–14]. Previous studies showed that prolonged hypothyroidism in BAT can induce effects similar to cold exposure [9,10,14,15], resulting in increased ACOX1 activity, gene expression and immunoexpression in BAT [12,16]. Further, hypothyroidism increases total ACOX activity in rat BAT [17]. Given that lipid breakdown requires the combined actions of distinct mitochondrial and peroxisomal β-oxidation systems, cooperation between peroxisomes, mitochondria and lipid bodies to maintain cell lipid homeostasis is to be expected [18–26].

Our previous data showed that hypothyroidism induced peroxisomal proliferation in BAT [27]. We also demonstrated the presence of heterogeneous population of peroxisomes in brown adipocytes, regarding a way of peroxisomal biogenesis and maturation rate [27], and their structural associations with mitochondria and lipid bodies (MPLB units) [28]. In this study, we set out to define if the structural association of peroxisomes with mitochondria, lipid bodies, or both are aligned with their functional compartmentalization in brown adipocytes. Hence, ACOX1 and ACOX3 protein expression, tissue and cell localization and colocalization of both isoforms within BAT of methimazole-induced hypothyroid rats were examined.

## 2. Material and methods

### 2.1. Animals and experimental design

All procedures performed in this study were approved by the Ethics Committee for the treatment of experimental animals at the Faculty of Biology within the University of Belgrade and by the Veterinary Directorate of the Ministry of Agriculture and Environmental Protection of the Republic of Serbia (ethical approval code: 323-07-07505/20l5-05/4). Two-month-old Wistar rats (330 ± 30 g) were maintained under 22 ± 1°C and 12 h light/dark cycles with ad libitum access to standard pelleted food. Randomized animals were divided into four groups each consisting of eight animals. Three groups were treated with 0.04% methimazole (Methimazole crystalline M8506, Sigma Aldrich Chemie GmbH, Germany) in drinking water for 7, 15, 21 days, respectively; animals in the fourth group served as control—euthyroid group (drinking tap water). After 21 days, animals were sacrificed using a decapitator (Harvard Apparatus, Holliston, MA, USA). Interscapular BAT (iBAT) was isolated and used for further analysis.

### 2.2. Western blotting

The left part of isolated rat BAT was used for Western blotting. Eight samples from each experimental group were pooled by three + three + two to obtain three samples per group. Thereafter, protein content was estimated [29]. Primary antibodies were used against ACOX1 (1 : 1000, ab-184032; Abcam, Cambridge, UK), β-actin (1 : 2000, ab-8226; Abcam, Cambridge, UK) and ACOX3 (1 : 500, sc-373977; Santa Cruz Biotechnology, Texas, USA). Immunoreactive bands in Western blots were quantified using ImageJ software (NIH, Bethesda, USA). The normalized target protein was averaged from three independent experiments and two representative bands per group were shown. The average value obtained in the euthyroid group was taken as 100%. Those from hypothyroid groups were expressed as percentages of the euthyroid group.

### 2.3. Light microscopy

Right part of the isolated BAT was fixed in 4% paraformaldehyde in phosphate buffer (pH 7.4). After fixation (12 h) the samples were washed in tap water overnight, dehydrated through a series of increasing concentrations of ethanol, cleared in xylene and then embedded in paraffin. Embedded tissue samples were cut by a rotating microtome (Reichert, Austria) to 5 μm thick sections, mounted to SuperFrost Plus microscopic slides, air-dried and used for immunohistochemistry. In order to simultaneously study ACOX1 and ACOX3 tissue and cell expression we used ‘mirror' technique. This method exploits the fact that serial sections were cut in order to produce adjoining iBAT sections appearing on the common surfaces in a mirror fashion. Subsequently, immunohistochemical staining for ACOX1 and ACOX3 was done.

### 2.4. Immunohistochemistry

After paraffin removal, rehydration, antigen retrieval by heat-induced method (high temperature and citrate buffer pH 6.0), and blocking of endogenous peroxidases, the serial sections of three animals per group were incubated with anti-ACOX1 (1 : 250, ab-184032) or anti-ACOX3 (1 : 250, sc-373977) primary antibodies overnight. An immunohistochemical reaction was performed using a commercial kit (DakoCytomation, LSAB+ System-HRP, Carpinteria, California, USA) with diaminobenzidine (DAB) as the chromogen. The reaction was stopped by rinsing with tap water. The contrast was achieved with Mayer's haematoxylin. After routine dehydration, permanent preparations were made by mounting in DPX medium (Mounting medium for histology, Sigma). All samples were viewed with a Leica DMLB light microscope using objectives: 20× (numerical aperture (NA) 0.40), 40× (NA 0.70) and 100× (NA 1.25). 8-bit images (resolution 2048 × 1536 pixels) were obtained via a Leica DFC295 digital camera (Leica Microsystems, Wetzlar, Germany) using Leica LAS AS v. 4.11 software.

### 2.5. Transmission electron microscopy

Small pieces of BAT immediately after isolation were fixed with 2% glutaraldehyde/2% paraformaldehyde in 0.1 M Sørensen phosphate buffer (PB, pH 7.2) for 1 h at 4°C. After fixation, the tissue was washed in PB and then preincubated in 0.1% 3,3′-diaminobenzidine (DAB) in PB for 30 min, for peroxisomal staining [30]. The preincubation medium also contained 0.01% H_2_O_2_ and was incubated for 1 h at 37°C. After washing in PB the tissue was post-fixed in 2% osmium tetroxide in the same buffer, then routinely dehydrated using increasing concentrations of ethanol and embedded in Araldite. Ultra-thin sections of BAT were obtained using a Leica UC6 ultramicrotome (Leica Microsystems, Wetzlar, Germany), mounted on nickel grids, air-dried and used for immunogold labelling.

### 2.6. Immunogold labelling

After antigen retrieval in 10 mM citrate buffer and incubation with 5% bovine serum albumin (BSA) in Tris-buffered saline/0.1% Tween 20 (TBS-T) for 1 h at room temperature, grids were incubated overnight at 4°C with mix of primary anti-ACOX1 (1 : 50, ab-184032) or anti-ACOX3 (1 : 50, sc-373977) antibodies. After rinsing in TBS-T, grids were incubated with 20 nm gold-conjugated anti-rabbit secondary antibody for ACOX1 (1 : 20, ab-27237) and 10 nm gold-conjugated anti-mouse secondary antibody for ACOX3 (1 : 20; ab-27241), for 1 h at room temperature, rinsed in TBS-T and double distilled water, air-dried and examined with a Philips CM12 transmission electron microscope (Philips/FEI, Eindhoven, The Netherlands) equipped with a SIS MegaView III digital camera (Olympus Soft Imaging Solutions, Münster, Germany). Images (8-bit, resolution 1376 × 1032 px) were obtained by iTEM 5.0 software. To test the specificities of the antibodies used in immunogold labelling, we performed both positive and negative controls. For the positive control we used ultra-thin sections of renal cortex tissue. We omitted the primary antibody when staining BAT sections for the negative control.

For quantification of ACOX1, ACOX3 and ACOX1/ACOX3 immunopositive peroxisomes, we analysed all brown adipocytes from the serial tissue cross-sections of three animals per group. The distribution of an absolute number of immunopositive peroxisomes per group is shown on the charts in figures 4 and 5, and representative, randomly chosen images are shown in figure 3.

### 2.7. Statistics

All the obtained data were analysed by GraphPad Prism v. 8.4.3. Normality within any given distribution was tested by D'Agustino and Pearson. If normality criteria were met, a one-way ANOVA with a *post hoc* multiple comparison test was performed. If normality criteria were not met, a Kruskal–Wallis non-parametric test was performed. Results are expressed as mean ± s.e.m. of obtained values. Significances were set at *p* < 0.05.

## 3. Results

### 3.1. Hypothyroidism alters ACOX1 and ACOX3 protein expression in BAT

**Figure 1.**
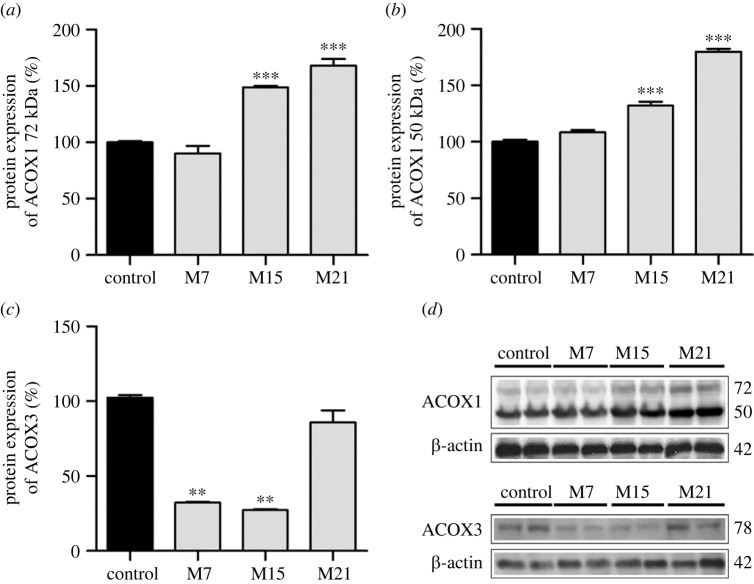
Protein expression of ACOX1 72 kDa (*a*), ACOX1 50 kDa (*b*), and ACOX3 (*c*) in rat BAT in control (black bars) and hypothyroid groups (grey bars) treated with methimazole for 7 (M7), 15 (M15) and 21 (M21) days. ACOX protein levels are expressed as a percentage of the control (set at 100%). Representative Western blot of three independent trials are shown (*d*). Data are represented as mean ± s.e.m. **p < 0.01; ***p < 0.001.

In order to access ACOX1 and ACOX3 protein expression within BAT of methimazole-induced hypothyroid rats, Western blot of both isoforms was performed (figure 1). Blot analysis revealed an increased protein expression of ACOX1 on day 15 of hypothyroidism and it remained high until the end of treatment (day 21). Since ACOX1 antibody shows two bands corresponding to 72 and 50 kDa at the Western blot [31,32], both were observed and showed the same changes (figure 1*a,b,d*). ACOX3 protein expression decreased after 7 days but returned to the control level on day 21 of hypothyroidism (figure 1*c,d*).

### 3.2. Tissue distribution of ACOX1 mirrors ACOX3 in a harlequin-like and time-dependent manner

**Figure 2.**
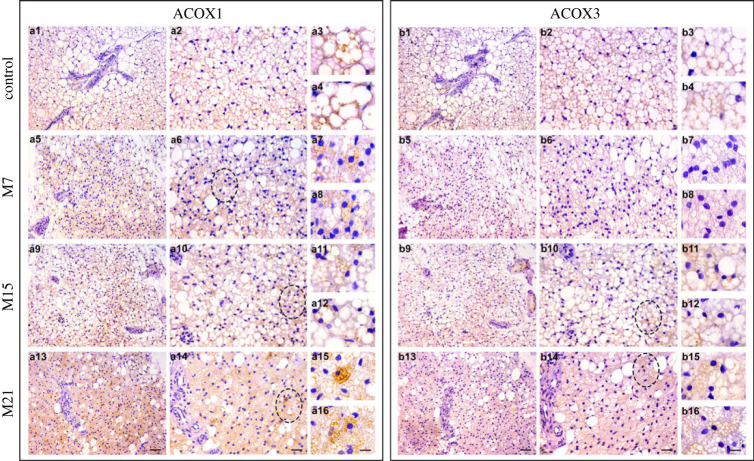
Immunohistochemical labelling of ACOX1 (*a*1–a16) and ACOX3 (*b*1–*b*16) in serial paraffin sections of BAT of euthyroid (*a*1–*a*4, *b*1–*b*4) and hypothyroid groups treated with methimazole for 7 (*a*5–*a*8, *b*5–*b*8), 15 (*a*9–*a*12, *b*9–*b*12) and 21 (*a*13–*a*16, *b*13–*b*16) days, respectively. Ellipse marked heterogeneous expression of ACOX1 and ACOX3 in different brown adipocytes within a tissue (Harlequin effect). Scale bars: *a*13, *b*13, 100 µm; *a*14, *b*14, 50 µm; *a*16, *b*16, 20 µm.

After revealing the protein expression pattern of ACOX1 and ACOX3 in BAT, we further studied the tissue distribution of these isoforms and performed their immunohistochemical labelling in mirror sections of BAT (figure 2). The method exploits the fact that tissue in adjoining sections appears on the common surfaces in a mirrored fashion. In one of the BAT sections, ACOX1 staining was performed, in the other section, ACOX3 staining took place.

By contrast to a relatively homogeneous distribution present in euthyroid control, the majority of ACOX1-positive brown adipocytes were distributed within tissue in a Harlequin-like manner over the course of hypothyroidism (figure 2*a*5–*a*16), (figure 2*a*1, *a*2). In brown adipocyte cytoplasm (figure 2*a*, insets), ACOX1 positive reaction appeared granular and mainly located around lipid bodies.

The majority of ACOX3-positive brown adipocytes on days 15 and 21 were distributed within tissue in a Harlequin-like manner (figure 2*b*9–*b*16), contrary to a relatively homogeneous distribution present in euthyroid control and on day 7 (figure 2*b*1–*b*8). Similarly to ACOX1, ACOX3 positive reaction appeared granular and distributed around lipid bodies but to a lesser extent than ACOX1 (figure 2*b*7–*b*16). Simultaneous analysis of BAT mirror sections revealed partial tissue colocalization of ACOX1 and ACOX3, both in euthyroid control and hypothyroid groups (figure 2).

### 3.3. ACOX-positive peroxisomes have specific localization patterns in brown adipocytes

**Figure 3.**
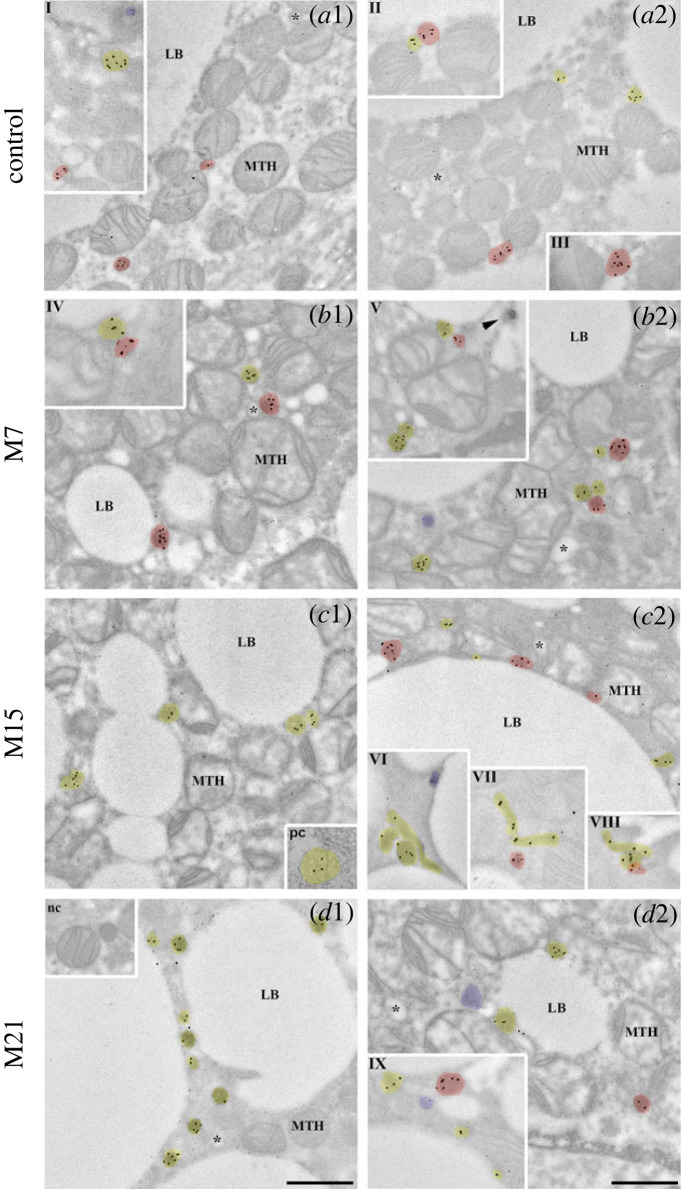
Immunogold labelling of ACOX1 and ACOX3 on ultrathin BAT sections with pre-stained peroxisomes (using DAB). Labelled peroxisomes from euthyroid control (*a*1, *a*2) and hypothyroid groups treated for 7 (M7, *b*1, *b*2), 15 (M15, *c*1, *c*2) and 21 days (M21, *d*1, *d*2) are indicated by colours: single ACOX1-positive peroxisomes, yellow; single ACOX3-positive peroxisomes, blue; double ACOX1/ACOX3-positive peroxisomes, red. In addition, positive (renal cortex cell, *c*1, inset pc) and negative (brown adipocytes, *d*1, inset nc) controls are shown. Mitochondria (MTH); lipid body (LB); endoplasmic reticulum (*); immunonegative peroxisome (not contain any ACOX isoform, ►). Size of gold particles: ACOX1, 20 nm; ACOX3, 10 nm. Scale bars 1 µm. Magnification for all electron micrographs 17 000× and all insets 28 000×.

**Figure 4.**
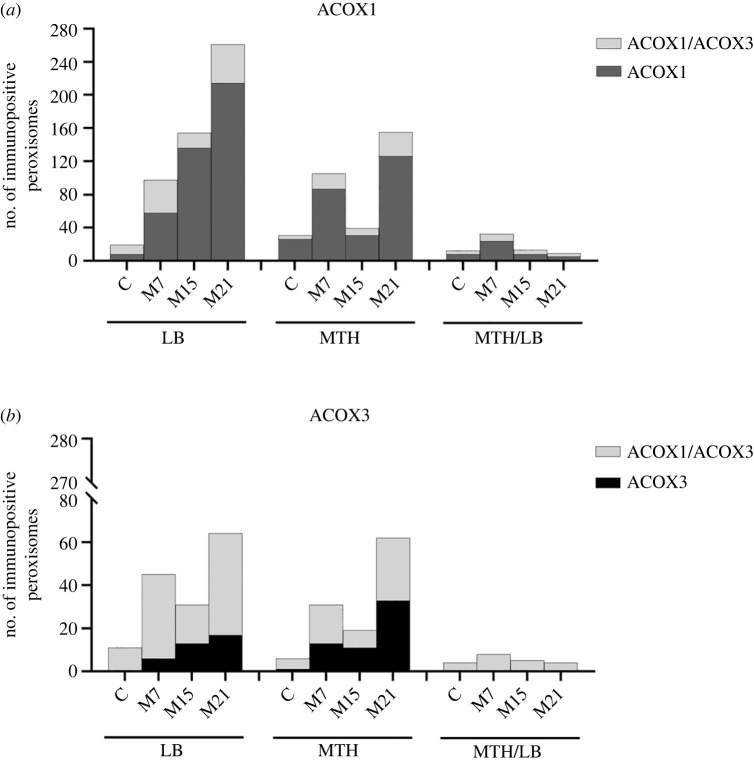
Distribution of: (*a*) single ACOX1-positive and double ACOX1/ACOX3-positive, and (*b*) single ACOX3-positive and double ACOX1/ACOX3-positive peroxisomes localized closely adjacent to lipid bodies (LB), mitochondria (MTH) and to mitochondria and lipid bodies together (MTH/LB) in brown adipocytes, within euthyroid control (C) and hypothyroid groups (M7, M15, M21). Bars represent the absolute number of single or double immunopositive peroxisomes per group.

**Figure 5.**
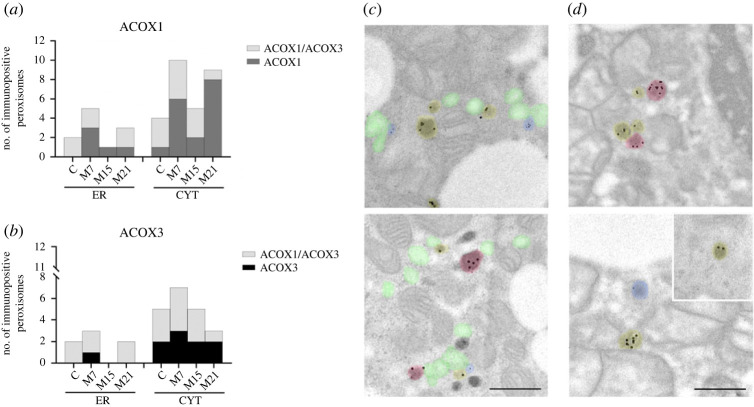
Distribution and representative electron micrographs of: (*a*,*c*,*d*) single ACOX1-positive (yellow) and double ACOX1/ACOX3-positive (red) peroxisome, and (*b*,*c*,*d*) single ACOX3-positive (blue) and double ACOX1/ACOX3-positive (red) peroxisomes localized closely adjacent to the endoplasmic reticulum (ER, green) and in the cytoplasm (CYT), in brown adipocytes within euthyroid control (C) and hypothyroid groups (M7, M15, M21). Bars represent the absolute number of single or double immunopositive peroxisomes per group (*a*,*b*). Magnification for all electron micrographs 17 000×. Scale bars 1 µm.

We further analysed ACOX1 and ACOX3 localization patterns and their colocalization within peroxisomes of single brown adipocytes over the course of hypothyroidism. Immunogold labelling of BAT ultra-thin sections specifically DAB pre-stained for peroxisomal localization was used. Different distributions of ACOX-negative, single ACOX1-, single ACOX3- and double ACOX1/ACOX3-positive peroxisomes, and different associations of these peroxisomes with lipid bodies (LB), mitochondria (MTH) or both (MTH/LB), and smooth endoplasmic reticulum (ER) were identified within different groups (figures 3–5).

The highest number of positively labelled peroxisomes was localized closely adjacent to the LB and MTH (figures 3 and 4). The lowest number of positively labelled peroxisomes was localized closely adjacent to the ER (figures 3 and 5).

Single ACOX1-positive peroxisomes were predominantly present closely adjacent to LB with a linear increase in number over the course of hypothyroidism (figures 3 and 4*a*). Most of the ACOX1 positive peroxisomes were single ACOX1-positive, although the number of double ACOX1/ACOX3-positive peroxisomes was substantial, and also increased in hypothyroidism (figures 3 and 4*a*). In the case of double ACOX1/ACOX3-positive peroxisomes, this increase was biphasic with a temporary decrease on day 15 of hypothyroidism (figures 3*c*1, *c*2 and 4*a*). A similar trend was observed in the case of single ACOX1-positive peroxisomes closely adjacent to MTH, although the number of positive peroxisomes was somewhat lower, and, importantly, the increases in ACOX1-positive peroxisomes over the course of hypothyroidism were biphasic in both single ACOX1- and double ACOX1/ACOX3-positive peroxisomes (figures 3 and 4*a*). On day 15 of hypothyroidism, many elongated peroxisomal structures (preperoxisomal structures), were observed (figure 3*c*1, *c*2). The number of single ACOX1-positive peroxisomes closely adjacent to MTH/LB was expectedly lower, and increase in number of ACOX1-positive peroxisomes (both single ACOX1 and double ACOX1/ACOX3-positive peroxisomes) was noticeable only on day 7 of hypothyroidism (figures 3*b*1, *b*2 and 4*a*).

The number of ACOX1-positive peroxisomes localized in cytoplasm was lower compared with peroxisomes closely adjacent to LB and MTH but showed the same biphasic increase over the course of hypothyroidism (figures 3 and 5*a*). In the case of single ACOX1-positive peroxisomes localized closely adjacent to the ER, the results were quite different: not only were there a modest number of immunopositive peroxisomes, but patterns of single ACOX1- and double ACOX1/ACOX3-positive peroxisomes were quite unique (figures 3 and 5*a*). On day 15 of hypothyroidism only single ACOX1-positive peroxisomes localized closely adjacent to the ER were present, while on other points of hypothyroidism, on days 7 and 21, we found both single ACOX1- and double ACOX1/ACOX3-positive peroxisomes (figures 3 and 5*a*).

In the terms of localization in brown adipocytes, single ACOX3-positive peroxisomes showed a somewhat similar pattern to that of single ACOX1-positive peroxisomes. Like ACOX1, ACOX3-positive peroxisomes were predominantly adjacent to the LB and MTH and showed increase in number over the course of hypothyroidism, and this increase was biphasic with an exception of single ACOX3-positive peroxisomes closely adjacent to LB where increase was linear (figures 3 and 4*b*). However, unlike ACOX1, ACOX3 was predominantly present in the peroxisomes also expressing ACOX1 (double ACOX1/ACOX3-positive peroxisomes), not as single ACOX3-positive peroxisomes (figures 3 and 4*b*). Further, there was no single ACOX3-positive peroxisome presence closely adjacent to MTH/LB (figure 4*b*).

Like ACOX1, single ACOX3-positive peroxisomes exhibited low presence in cytoplasm and closely adjacent to ER (figures 3 and 5*b*). In cytoplasm, single ACOX3-positive peroxisomes increase was apparent only on day 7 of hypothyroidism. Closely adjacent to the ER, ACOX3-positive peroxisomes showed exclusive pattern: absence on day 15, single ACOX3-positive peroxisomes presence on day 7, and double ACOX1/ACOX3-positive peroxisomes presence in euthyroid control and on days 7 and 21 of hypothyroidism (figure 5*b*).

## 4. Discussion

Herein, we demonstrated that hypothyroidism affects both ACOX1 and ACOX3 protein expression and induces their distinct tissue and cell heterogeneous localization/colocalization in BAT.

ACOX1 is the dominant isoform in BAT, that linearly increases over the course of hypothyroidism. This could have been a consequence of a transcription/translation switch that favours ACOX1 expression over the course of hypothyroidism. Namely, it was shown that hypothyroidism alters lipid metabolism in favour of long, branched fatty acids serving as an ACOX1 substrate [33–35]. Furthermore, in BAT, hypothyroidism acts in a similar way as cold exposure [12,16], inducing thermogenesis, i.e. increasing gene, immunoexpression and ACOX1 activity [9,10,14,15]. This further emphasizes the important role of peroxisomes not only in lipid metabolism but also in thermogenesis as mitochondria's helpers during oxidation processes. In this way peroxisomes and mitochondria jointly contribute to the overall BAT thermogenesis.

So far, there are no published data regarding ACOX3's presence and its function in BAT or its relation with ACOX1. We demonstrated ACOX3 presence in euthyroid rat BAT and its temporarily decreased levels in the early days of hypothyroidism. A plausible explanation for the reduced ACOX3 expression in the early days of hypothyroidism may lay in the fact that hypothyroidism affects systemic lipid metabolism [36], which is reflected in the change of ACOX substrates for oxidation in BAT in favour of ACOX1.

Given observed patterns of ACOX1 and ACOX3 protein expression, we further studied tissue and cell distributions of these isoforms on BAT mirror sections. Distinctive ACOX1 and ACOX3 presence within brown adipocytes is a pattern described as a Harlequin effect. The Harlequin effect implies the heterogeneous expression of both isoforms at the tissue level, reflecting hypothyroidism-induced peroxisomal proliferation in BAT. We already described peroxisomal diversity in brown adipocytes regarding specific DAB staining, catalase positivity or different pathways of biogenesis and their size within specific ‘pearls on strings' structures [27]. This heterogeneity is a result of their different localizations in the cytoplasm and their association with other organelles, or/and different enzyme content and subsequently different functions in the cells [37,38].

This further raises the question of whether the appearance of tissue Harlequinism—peroxisomal heterogeneity—is a consequence of ACOX1 and ACOX3 structural and functional compartmentalization in single brown adipocytes. Using ACOX1 and ACOX3 immunogold labelling we found four different peroxisomal populations in brown adipocytes with respect to their immunopositivity: single ACOX1-positive peroxisomes, single ACOX3-positive peroxisomes, double ACOX1/ACOX3-positive peroxisomes and (rarely) immunonegative peroxisomes.

Similar heterogeneous distribution of peroxisomes in the cells or tissue has been shown in regenerating rat liver [39], but also in adult rat liver, kidney and small intestine [40]. In addition, ‘peroxisomal mosaicism' has been demonstrated in patients with a mild form of Zellweger syndrome, due to the fact that peroxisomal biogenesis is less affected in some cells and more affected in other cells. It appears as a difference in distribution of either matrix proteins or both, matrix and peroxisomal membrane proteins [41].

In our model of hypothyroidism, mosaicism/peroxisomal heterogeneity was shown at two levels. One is ACOX1, ACOX3 and ACOX1/ACOX3 peroxisomal immunopositivity, which underscores high functional peroxisomal heterogeneity in brown adipocytes. Functional heterogeneity means that different populations of peroxisomes within one cell perform different functions, based on the high specificity of the populations toward the substrate. The other level is peroxisomal structural association with mitochondria and/or lipid bodies, allowing close interorganellar communication and cooperation, and underlining peroxisomal functional compartmentalization in the cell.

It is of notable interest that hypothyroidism changed the localization preferences of single ACOX1-positive, single ACOX3-positive or/and double ACOX1/ACOX3-positive peroxisomes. The highest number of single ACOX1-positive, single ACOX3-positive and double ACOX1/ACOX3-positive peroxisomes was localized closely adjacent to lipid bodies and mitochondria. Localization of ACOX-positive peroxisomes closely adjacent to lipid bodies and mitochondria indicated close functional cross-talk between structurally associated organelles. It has been well documented that peroxisomes and mitochondria cooperate in processes such as β-oxidation of fatty acids and antioxidative defence [20,22,42] and that peroxisome-derived lipids regulate thermogenesis by mediating cold-induced mitochondrial dynamics [18]. Further, consistent with the close relationship between peroxisomes and mitochondria, recent studies have shown peroxisomal *de novo* biogenesis from mitochondria [27,43–46]. Peroxisomes are also associated with lipid bodies in different experimental models [24–26,47]. Moreover, Bartz *et al.* showed the presence of bidirectional lipid trafficking between peroxisomes and lipid bodies [23], suggesting coordinated regulation of lipid metabolism and energy balance by peroxisomes and lipid bodies [21,23]. Under various metabolic conditions, contacts between lipid bodies and mitochondria serve as sites for both lipogenesis and lipolysis [48].

We also showed that a low number of single ACOX1-positive and double ACOX1/ACOX3-positive peroxisomes were localized closely adjacent to mitochondria and lipid bodies at the same time, while single ACOX3-positive peroxisomes were absent from this organellar arrangement. Immunopositive peroxisomes were dominant at the beginning of hypothyroidism (on day 7). We previously demonstrated that hypothyroidism induced close structural association of mitochondria, peroxisomes, and lipid bodies—MPLB units [28]. Thus, the structural unity of the major functional actors in brown adipocytes, followed by a specific antioxidative defence enzyme localization pattern, suggests a unique functional syncytium that supports the redox-dependent changes induced by hypothyroidism [28]. Considering the ACOX-related functional heterogeneity of peroxisomes in brown adipocytes, we assume that peroxisomes associated with mitochondria and lipid bodies were more dedicated to the scavenging of H_2_O_2_ than to the β-oxidation of fatty acids. The absence of single ACOX3-positive peroxisomes in this specific association of organelles further supports this assumption. It is known that peroxisomes can reduce the H_2_O_2_ they produce themselves, but also extra-peroxisomal H_2_O_2_ [49–51]. Therefore, the potential involvement of peroxisomes in scavenging extra-peroxisomal H_2_O_2_ could be a reason for the low immunoexpression of ACOX1 and ACOX1/ACOX3, but also for the absence of ACOX3 in peroxisomes when they associate with mitochondria and lipid bodies at the same time. This is consistent with the findings of Gao *et al*. [52] that a mixture of long-chain saturated fatty acids and unsaturated fatty acids stimulate the production of reactive oxygen species in adipocytes and lead to mitochondrial dysfunction [52]. This unique functional cluster containing peroxisomes highlights the importance of peroxisomes in maintaining metabolic homeostasis and controlling energy expenditure within brown adipocytes.

The lowest number of positive peroxisomes was localized closely adjacent to the endoplasmic reticulum and in the cytoplasm. We assume that the positive peroxisomes localized in the cytoplasm represented a transition form of peroxisomes that were in the process of being transported to the vicinity of their functional partner organelles. We can also assume that immunopositive peroxisomes localized in the cytoplasm preferably oxidize uptaken fatty acids. This is supported by the fact that not only intracellular fatty acids released from lipid bodies but also extracellular lipids are necessary for thermogenesis in BAT [53,54]. Increased uptake of triglycerides by skeletal muscle in hypothyroid rats has been observed [55]. In addition, increased uptake of triglycerides by BAT upon cold exposure has been observed [56].

A very low number of positive peroxisomes localized closely adjacent to the endoplasmic reticulum may point to their origin from the endoplasmic reticulum and their rapid functional maturation, which parallels their structural maturation [27]. Still, we found a similarity between ACOX1 and ACOX3 localization patterns. In that process of rapid functional maturation, some peroxisomes were still in structural association with the endoplasmic reticulum from which they bud. In our earlier work, we also hypothesized that some peroxisomes in brown adipocytes remain structurally connected to the organelle from which they originate for easier communication [27].

## 5. Conclusion

The presence of different ACOX isoforms in peroxisome is physiologically important regarding the substrate specificity and selectivity handling for the peroxisomal β-oxidation. Many metabolic disorders are manifesting as disturbances in lipid metabolism that may lead to excessive lipid accumulation or lipotoxicity [57–59]. Hence, changes in ACOX content, localization and colocalization in peroxisome may be useful in preventing metabolic disorders or other ailments since recent studies suggested ACOX1 as a potential therapeutic biomarker in colon cancer [60,61]. Thus, an important question arises regarding peroxisomal functional heterogeneity in tissues and its contribution to the prevention of lipid disturbances and other disorders.

We demonstrated that peroxisomal ACOX1 and ACOX3 localization and colocalization patterns entirely mirrored heterogeneous peroxisomal biogenesis pathways and structural compartmentalization, e.g. associations. Specific association of single ACOX1-positive, single ACOX3-positive and double ACOX1/ACOX3-positive peroxisomes with mitochondria and/or lipid bodies creates distinct functional heterogeneity of peroxisomes and drives their functional compartmentalization in rat brown adipocytes. This study provides a better understanding of both heterogeneous peroxisomal functions as well as the heterogeneous peroxisomal associations with other organelles (mitochondria and lipid bodies), shedding light on the roles of organellar cross-talk in lipid metabolism as a basis of thermogenesis in brown adipose tissue.

## Data Availability

The datasets supporting this article have been uploaded as part of the electronic supplementary material [62].
